# Long-term application of agronomic management strategies effects on soil organic carbon, energy budgeting, and carbon footprint under rice–wheat cropping system

**DOI:** 10.1038/s41598-023-48785-z

**Published:** 2024-01-03

**Authors:** R. K. Naresh, P. K. Singh, Rajan Bhatt, Mandapelli Sharath Chandra, Yogesh Kumar, N. C. Mahajan, S. K. Gupta, Nadhir Al-Ansari, Mohamed A. Mattar

**Affiliations:** 1 Department of Agronomy, Sardar Vallabhbhai Patel University of Agriculture & Technology, Meerut, UP India; 2 Director Extension Education, Sardar Vallabhbhai Patel University of Agriculture & Technology, Meerut, UP India; 3 Krishi Vigyan Kendra, Amritsar, Punjab Agricultural University, Ludhiana, Punjab India; 4 AICRP On Integrated Farming System, Professor Jayashankar Telangana State Agricultural University, Rajendranagar, Telangana India; 5 Department of Soil Science & Agricultural Chemistry, Sardar Vallabhbhai Patel University of Agriculture & Technology, Meerut, UP India; 6 Institute of Agricultural Science, Department of Agronomy, Banaras Hindu University, Varanasi, U. P India; 7 Department of Agronomy, Bihar Agricultural University Sabour, Bhagalpur, Bihar India; 8 Department of Civil, Environmental and Natural Resources Engineering, Lulea University of Technology, 97187 Lulea, Sweden; 9 Prince Sultan Bin Abdulaziz International Prize for Water Chair, Prince Sultan Institute for Environmental, Water and Desert Research, King Saud University, 11451 Riyadh, Saudi Arabia; 10 Department of Agricultural Engineering, College of Food and Agriculture Sciences, King Saud University, 11451 Riyadh, Saudi Arabia

**Keywords:** Sustainability, Carbon cycle

## Abstract

In the plains of western North India, traditional rice and wheat cropping systems (RWCS) consume a significant amount of energy and carbon. In order to assess the long-term energy budgets, ecological footprint, and greenhouse gas (GHG) pollutants from RWCS with residual management techniques, field research was conducted which consisted of fourteen treatments that combined various tillage techniques, fertilization methods, and whether or not straw return was present in randomized block design. By altering the formation of aggregates and the distribution of carbon within them, tillage techniques can affect the dynamics of organic carbon in soil and soil microbial activity. The stability of large macro-aggregates (> 2 mm), small macro-aggregates (2.0–2.25 mm), and micro-aggregates in the topsoil were improved by 35.18%, 33.52%, and 25.10%, respectively, over conventional tillage (0–20 cm) using tillage strategies for conservation methods (no-till in conjunction with straw return and organic fertilizers). The subsoil (20–40 cm) displayed the same pattern. In contrast to conventional tilling with no straw returns, macro-aggregates of all sizes and micro-aggregates increased by 24.52%, 28.48%, and 18.12%, respectively, when conservation tillage with organic and chemical fertilizers was used. The straw return (aggregate-associated C) also resulted in a significant increase in aggregate-associated carbon. When zero tillage was paired with straw return, chemical, and organic fertilizers, the topsoil's overall aggregate-associated C across all aggregate proportions increased. Conversely, conventional tillage, in contrast to conservation tillage, included straw return as well as chemical and organic fertilizers and had high aggregate-associated C in the subsurface. This study finds that tillage techniques could change the dynamics of microbial biomass in soils and organic soil carbon by altering the aggregate and distribution of C therein.

## Introduction

Nearly two-thirds of the carbon used in agriculture is stored in the soil. In the upper 1m of SOC, around 1500 Pg (1 Pg = 10^–9^ mg = 10^–12^ g) of carbon have been retained^1^. The remaining (560 Pg) terrestrial carbon is stored in plant biomass^2^. The seas act as a reservoir for carbon because of its capacity to cature and store carbon (38,000 Pg) and atmosphere also has a higher capacity to store carbon (750 Pg), when compared with the soil^2^ according to Stockmann et al.^1^. Over the past 35 years, carbon dioxide (CO2) emissions from anthropogenic sources, such as the burning of fossil fuels and the manufacture of cement, have increased. In the 1980s, anthropogenic carbon emissions peaked at 6 Pg yr^–1^^3^. In 2014, man-made carbon emissions rose by 10 Pg yr^–1^^4^. As carbon sinks, soils can contribute to reducing atmospheric carbon dioxide levels and the greenhouse effect^5^. Over 10 million hectares (M ha) of the Indian Indo-Gangetic Plains (IGPs) are covered by the rice–wheat crop rotation, which accounts for 85% of the country's grain production and provides a foundation for millions of food consumers and producers. Issues connected to a loss in soil quality have been made worse by a lack of or insufficient use of organic amendments and the removal of agricultural waste^6^, as well as by widespread monoculture use^7^ and imbalanced use of synthetic fertilizers^8,9^.

However, how much amount of carbon (C) can be stored by the soils is depends on the amount of carbon that can be taken up by the plants during production process and how much is exported, both of which are controlled by microbial breakdown^8^. The management strategies employed to ensure greater C returns to the soil are projected to lead to a net rise of Total Organic Carbon (TOC)^10^. Maintaining TOC reserves is essential for increasing agricultural sustainability and lowering carbon emissions to prevent global warming^8^. The enhancement of fertile soil, the maintenance of the soil's structure, the reduction of CO_2_ emissions, and the promotion of microbial diversity are all closely correlated with farmland SOC sequestration^11–13^. The SOC content of Chinese farming soil, however, is frequently poor, falling more than 30% below the global average and more than 50% below that of Europe^15^, according to Chen et al.^14^. As a result, increasing the SOC content of cultivated soil has received significant attention in agricultural research. Human activities, as opposed to regional natural factors like weather and soil characteristics, have a greater impact on the variance in the agriculture SOC stock, according to Tang et al.^16^ and Gonçalves et al.^17^. By improving soil aggregate integrity and increasing SOC intake, these interventions primarily increase cropland's SOC content^19^. No-tillage and straw return are beneficial for building SOC, although some studies believe they may potentially reduce crop production^20^. Soil microbial biomass is one potential measure of soil quality that responds swiftly to management- and environment-induced changes^21^. All crop wastes undergo at least one transfer of carbon from one carbon pool to another due to soil microbial biomass and ultimately carbon dioxide loss^22^. Crop residue that hasn't completely broken down and is remaining in the soil aids in carbon sequestration^23^. Thevenot et al.^24^ claim that how rice leftovers degrade is directly connected to their quality, with non-cellulosic polysaccharides and hemicelluloses degrading quickly in the first stage and steady C in both cellulose and lignin-degrading slowly in the second. Organic soil C is a heterogeneous mixture of labile and stable (i.e., recalcitrant) organic C pools, based on variable turnover rates^25^. Labile C fractions operate as sensitive markers of soil management-induced changes in the TOC pool in the short and medium term^26^. These pools have quicker turnover rates than TOC and are a better indicator of soil strategy actions^27^.

The main source of C consumption has been rice straw (RS), which is produced in large quantities by rice–wheat cropping system^28^. The next wheat crop benefited from the use of leftover rice in the soil^29^. However, using a normal tillage method has led to a serious issue with soil deterioration because agricultural wastes are burned on-site^30^. The most practical management strategy for increasing soil fertility and agricultural output, according to Yadvinder-Singh and Sidhu^31^, has been the use of rice straw, a crucial organic C source that also provides nutrients for the best crop yields. The RS addition increased the TOC pool by 13% and the labile components by 42% as compared to using chemical fertilizers alone^33^. Without tillage and straw return, as well as a number of organic and inorganic fertilizer management choices, were predicted to increase soil organic carbon fractions, energy budgeting, and reduce carbon footprint in the crop rotation systems of rice–wheat. As for the aforemtioned facts, the long term study was conducted to evaluate the agronomic management practices on soil organic carbon, energy budgeting, and carbon footprint in under rice–wheat cropping system in indo gangatic plains of North western Uttar pradesh, India.

## Materials and methods

### Experimental site and climate

At the Crop Research Center farm of the Sardar Vallabhbhai Patel University of Agriculture & Technology, Meerut, Uttar Pradesh, (29° 04′N and 77°42′E) in the IGPs of north-western India, a field experiment on the cropping system of rice and wheat was commenced in 2000. The soil contains clay, silt and sand of about 128 g, 155 g and 715 g per kilogram, which comes under sandy loam soil (0–15 cm) according to USDA classification, and it was categorized as *Typic Ustochrept*. The region normally experiences a subtropical, semi-arid environment with an average annual rainfall of 700 mm, of which 75–80% fall between July and August, and the remaining 20–25% fall between December and February. The average monthly temperature lies in the range of 12.8 to in general. At the beginning of the experiment, the surface soil had an electrical conductivity, E.C.1:2 (soil: water suspension) = 0.34 dS m^–1^, a pH1:2 (soil: water suspension) of 8.4, 3.91 g TOC kg^–1^, 22.4 mg available P kg^–1^, and 134.4 mg available K kg^–1^. Wet digestion was used to compute the total organic C^34^, available P using the method of ascorbic acid blue colour method using a spectrophotometer, in this method was found to be accurate for determining P in soil extracts. This method is based on reduction of the ammonium molybdiphosphate complex by ascorbic acid in the presence of antimony. The color produced is stable for 24 h. It is less subject to interfering substances than are methods involving reduction by SnCl_2_, and available K estimated by extracting the soil using normal ammonium acetate at a pH of 7.0, then calculating K using a flame photometer.

### Experimental planning and field supervision

The investigation, which began in earnest in 2000, consisted of a total of fourteen treatments that combined various tillage techniques, fertilization methods, and whether or not straw return was present (Table 1). Three replications of each treatment were included, and a single-factor random block design was used to set it up. Each plot had a 240 m^2^ space and measured 30 m long by 8m broad. Plastic film was used to divide the irrigation/drainage ditch ridges between the blocks and the plot ridges. Rotary tillage was done at a depth of 20 cm, while traditional tillage was 30 cm deep. In the straw return plots, both rice and wheat stalk biomass was restored to the soil under the T_3_ (Zero-tillage clubbed with straw return and chemical fertilizer), T_5_ (Zero-tillage clubbed with straw return with organic fertilizer and chemical fertilizer), T_7_ (Rotary tillage clubbed with straw return and chemical fertilizer), T_9_ (Rotary tillage clubbed with straw return with organic fertilizer and chemical fertilizer), T_11_ (Conventional tillage clubbed with straw return and chemical fertilizer), and T_13_ (Conventional tillage clubbed with straw return with organic fertilizer and chemical fertilizer) treatments. The portion of the plots without a straw return that is above ground In both the rice and wheat seasons, 40,000 kg hm^–2^ (kilogram per square hectometer) of organic fertilizer were applied. In each agricultural season, base fertilizers such as straw, organic fertilizer, P fertilizer, and K fertilizer were applied once before planting. In the organic fertilizer, we used farm yard manure (FYM) and straw residue retain on the surface of the soil in zero till and same quantity of straw residue incorporate in conventional tillage as per the treatments. Base fertilizer and topdressing made up the two components of the N fertilizer. Before planting during the rice season, 50% of the N, 100% of the P, K, and organic fertilizers were put on each plot. After sowing, the leftover N is given in two equal amounts at 20 and 60 days. All of the P, K, and organic fertilizers as well as 50% of the N were treated before sowing during the wheat season. Equal amounts of the remaining N were added 30 and 60 days after seeding. Pest control and field management are done accordingly.

**Table 1:** Detailed treatments of the experiment.

Treatments	Cultivation management	Abbreviations
T_1_	Conventional tillage without straw return and without chemical fertilizer	“Control”
T_2_	Zero-tillage clubbed with chemical fertilizer	ZT + CF
T_3_	Zero-tillage clubbed with straw return and chemical fertilizer	ZT + R + CF
T_4_	Zero-tillage clubbed with organic fertilizer and chemical fertilizer	ZT + O + CF
T_5_	Zero-tillage clubbed with straw return with organic fertilizer and chemical fertilizer	ZT + R + O + CF
T_6_	Rotary tillage clubbed with chemical fertilizer	RT + CF
T_7_	Rotary tillage clubbed with straw return and chemical fertilizer	RT + R + CF
T_8_	Rotary tillage clubbed with organic fertilizer and chemical fertilizer	RT + O + CF
T_9_	Rotary tillage clubbed with straw return with organic fertilizer and chemical fertilizer	RT + R + O + CF
T_10_	Conventional tillage clubbed with chemical fertilizer	CT + CF
T_11_	Conventional tillage clubbed with straw return and chemical fertilizer	CT + R + CF
T_12_	Conventional tillage clubbed with organic fertilizer and chemical fertilizer	CT + O + CF
T_13_	Conventional tillage clubbed with straw return with organic fertilizer and chemical fertilizer	CT + R + O + CF
T_14_	Conventional tillage farmers practices	CTFP

### Soil sampling and processing

To assess the cumulative effects of 21 years of treatments on soil organic carbon (SOC) dynamics, we collected undisturbed soil cores (2–1/4" (5.7 cm) in diameter) from all treatment plots after the May 2021 wheat harvest, at depths of 0–15 cm and 15–30 cm. Composite samples were created from individual plot samples. These were divided: one part was refrigerated for analyzing biological parameters, and the other part was air-dried and sieved to remove aggregates larger than 5 mm. Sub-samples from both depth ranges were examined for soil agglomeration and SOC content, revealing the treatment impacts on soil structure and carbon dynamics.

### Particulate organic carbon (POC)

To obtain a fraction of Particulate Organic Matter (POM), 50 g of air-dried soil samples were immersed during the 30-min deionization process. The slurry was then placed in a 250-mm sieve housed on the inward side of a cylinder and vigorously agitated over 120 revolutions per minute within 50 10-mm glass beads. The 25-m-deep bottom sieve was used to capture the micro-aggregates that made it through the 250-m sieve. Coarse POM was the name given to the fraction on the 250-m sieve that was gritty in texture (POM and sand via 250 to 2000-m). To isolate the fine POM, using 25 ml of sodium hexametaphosphate solution at a concentration of 0.5 g/l and 12 4-mm glass beads, the aggregates that were still on the 25-m sieve (with sizes varying between 25 and 250 lm) were agitated for 18 h^35^.

### Microbial biomass carbon (MBC)

Using the chloroform fumigation and incubation method created by Ref.^36^, each soil sample was split into two subsamples for fumigated and non-fumigated treatments. The goal was to determine the soil microbial biomass (C and N). Field water capacity was adjusted to 55% moisture in the soil. Samples of soil weighing 30 g were fumigated with CHCl_3_ for 24 h at 25 °C to test for MBC. Following the removal of the CHCl_3_, each soil sample is incubated at 25°C for 10 days in a firmly closed Mason jar using glass vials containing 1.0 ml of 2 M NaOH. The amount of CO_2_-C flush emitted during fumigation is calculated using HCl titration. Equation (1) was used to calculate the MBC:

1 $$ {\text{MBC}}\,\left( {{\text{mg kg}}^{{{-}{1}}} } \right)\, = \,\left( {{\text{Fc}} - {\text{UFc}}} \right)/{\text{Kc}}{.} $$

Here, Fc has been the CO_2_ that has been evolved through fumigated soil, UFc is indeed the CO_2_ that has been evolved using unfumigated soil, and Kc is indeed a variable with a value of 0.41^37^.

### Microbial biomass nitrogen (MBN)

Microbial biomass N was calculated as a difference in N content in fumigated and non-fumigated sample (EN) using kEN coefficient (microbial biomass N = EN:kEN). The value of kEN = 0.54 was used to calculate microbial biomass N.

2 $$ {\text{MBN }}\left( {{\text{mg kg}}^{{{-}{1}}} } \right) \, = {\text{ E}}_{{\text{N}}} /{\text{k}}_{{{\text{EN}}}} , $$

where EN = (total N extracted from fumigated soils) − (total N extracted from non-fumigated soils) and kEN = 0.54^37^.

### Soil carbon content assessment

The quantity of soil organic carbon was determined by wet digestion using potassium dichromate and even a 3:2 H_2_SO_4_:85% H_3_PO_4_ digestion mixture inside a digestive unit fixed at 1200C for 2 h^34^. For the purpose of removing carbonate and bicarbonate, 3 ml of a solution containing 1 N HCl per g of soil was employed as a pre-treatment. The following calculation was employed to ascertain the level of SOC in the samples collected.

3 $$ {\text{SOC concentration }} = {\text{ Total C }}{-}{\text{ Inorganic C}}. $$

Profile SOC stock: For each of the five depths (0–15, 15–30, 30–60, 60–80, and 80–100 cm), the total SOC stock of the profile was calculated as Mg ha^–1^ by multiplying the SOC concentration (g kg^–1^) (obtained by SOC = LECO C-HCl C) by the bulk density (Mg m^–3^) and depth (cm), and by 10. We clearly indicate that the LECO C values are not derived as mentioned in this above formulae.

### Budgeting for carbon calculations

The governing formula were used to compute the carbon budgeting:

4 $${C}_{restoration}\left(\%\right)=\frac{{C}_{fert+org }\;or\; {C}_{fert}-{C}_{cont}}{{C}_{cont}}\times 100,$$

where C_fert + org_ represent C in Fertilizer + farm yard manure (FYM) treatments and C_fert_ and C_cont_ is the C in fertilizer and control treatments, respectively.

5 $${C}_{restoration}\left(Mg C {ha}^{-1}\right)=\frac{{C}_{fert+org }\;or\; {C}_{fert}-{C}_{cont}}{Year of experimentation}\times 100,$$

6 $${C}_{stabilization}\left(\%\right)=\frac{{C}_{fert+org }or {C}_{fert}}{{C}_{org}}\times 100,$$

where C_org_ represents C applied through organic material (i.e., FYM)

7 $${C}_{sequestered}\left(Mg C {ha}^{-1} soil\right)={SOC}_{current}-{SOC}_{initial}$$

where SOC current and SOC initial refer to the SOC stocks as of the start of the long-term experiment in 2020 and in 2000, respectively. Gains and losses in SOC stocks are indicated accordingly by positive and negative figures. Following Bhattacharyya et al.^38^, the following relationship was used to determine carbon retention efficiency (CRE):

8 $$CRE\left(\%\right)=\frac{{SOC}_{final}-{SOC}_{initial}}{ECI}\times 100,$$

In this scenario, SOC final and SOC initial stand for SOC (Mg ha^–1^) in the initial and final soils, respectively, while ECI is the projected cumulative C intake (Mg ha^–1^) to the soil between the first and final year of the experiment.

### Aggregate size distribution

Distribution of aggregate size was expressed as the structure coefficient (Ks), is calculated according to Shein et al.^39^:

9 $$Ks=a/b,$$

where a represents the weight percentage of aggregates 0.25–10 mm and b represents the weight percentage of aggregates < 0.25 mm and > 10 mm.

The distribution of particle sizes was measured by sieving and using the pipette method, with sodium pyrophosphate as a dispersing agent. Soil water retention was measured at matric potentials of -33 and -1500 kPa using a porous plate and pressure membrane apparatus.

### Analytical statistics

The Windows-based SPSS application was used for statistical analysis to determine the statistical validity of treatment outcomes (16.0, SPSS Inc., 1996). The means were compared using the least significant difference (LSD) of Duncan's Multiple Range Test (DMRT). Statistics consider a probability level of 5.0% to be significant.

## Results and discussion

### Different types of soil carbon

**Figure 1:**
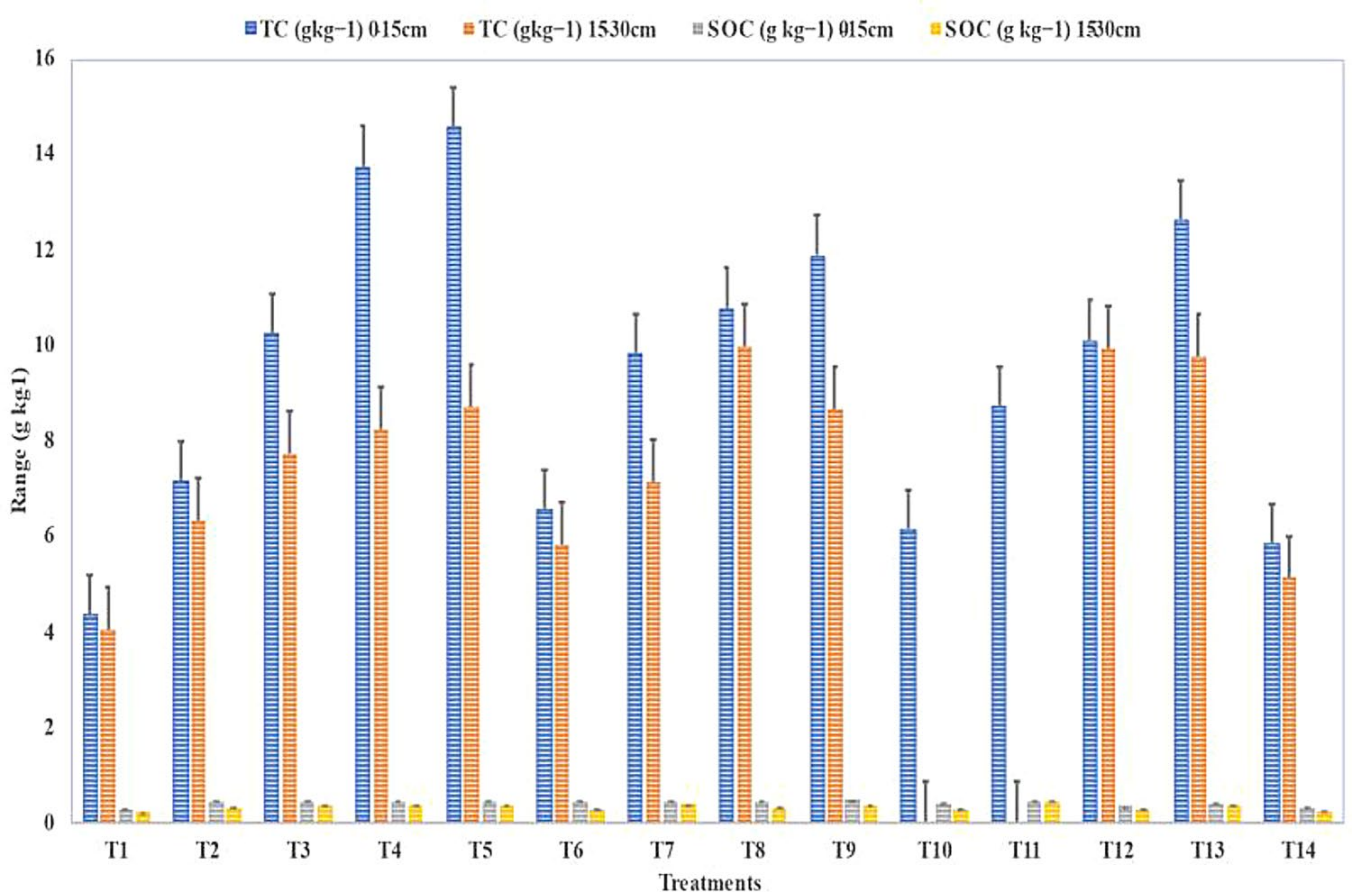
After 21 years of rice–wheat cultivation, soil total carbon (TC) and soil organic carbon (SOC) both topsoil and subsoil were measured under various treatments.

Figure 1 displays the impacts of a 21-year rice–wheat crop rotation using different crop farming techniques on soil carbon. Carbon levels in total (TC) and organic soil carbon levels (SOC) in topsoil on average for the subsurface soils were 9.42 g kg^−1^, 0.42 g kg^−1^, 7.63 g kg^−1^, and 0.34 g kg^−1^, respectively.

The mean levels of the 3-carbon types with zero tillage have been higher than those under conventional tillage as well as zero tillage. The TC and SOC concentrations were considerably lower than those with rotary tillage. When compared to top soil and traditional tillage without chemical fertilizers, the subsurface contents of TC and SOC follow similar trends (T_1_). In addition to tillage, changes in soil carbon concentration between the various treatments were also caused by straw return and organic fertilizer. Between 2000 and 2021, the average topsoil TC concentrations under the use of organic fertilizer (T_4_, T_8_, and T_12_) and straw return (T_3_, T_7_, and T_11_) were 16.83% and 19.78% higher than those under the use of chemical fertilizer alone (T_2_, T_6_, and T_10_).

### Aggregate size distribution

**Figure 2:**
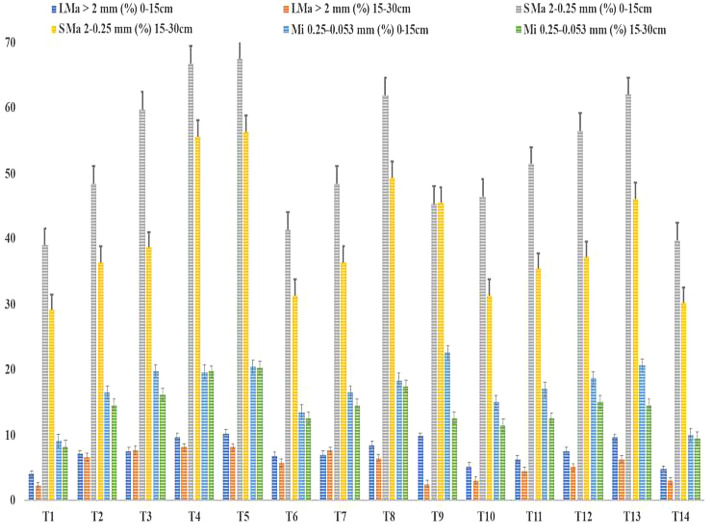
Over 21 years (2000–2021) of such rice–wheat crop rotation, big macroaggregates (LMa), tiny macroaggregates (SMa), and micro aggregates (Mi) in soil surface and subsurface with various treatments.

The proportionate percent of soil aggregates produced after wet sieving is shown in Fig. 2. The percentages of topsoil in big macro-aggregates (> 2 mm), tiny macro-aggregates (> 2.25 mm), as well as micro-aggregates (0.25 mm), were 10%, 50%, and 20%, respectively. Although less plentiful than topsoil, the subsurface constituents of the three varied aggregates showed comparable distribution patterns (Fig. 2).

During the period from 2000 to 2021, the treatment T_5_ recorded the highest number of big macroaggregates, tiny macroaggregates, and microaggregates at both depths and at 15 to 30 cm, followed by T_4_. In contrast, T_9_ recorded the highest number of micro-aggregates at 0 to 15 cm, followed by T_13_. The content of macroaggregates and microaggregates significantly increased because of zero-tillage with straw return and organic fertilizer, notably in the topsoil. As depicted in Fig. 2, compared to conventional tillage, zero-tillage (T_2_, T_3_, T_4_, and T_5_) increased the number of big macroaggregates (10.48%) and tiny macroaggregates (9.62%) (T_10_, T_11_, T_12_, and T_13_). The subsoil showed a similar pattern. Despite the same tillage, the surface and subsurface contained, in order of importance, the respective large and tiny macroaggregates: straw return > organic fertilizer > a sole use of chemical fertilizer application (Fig. 2). The number of large macroaggregates was particularly higher under zero-till clubbed with the return of straw plus organic fertilizers (T_5_), such values indicated 6.78%, 10.95%, and 28.57% greater than those with the sole use of chemical fertilizers (T_2_, T_6_, and T_10_). The number of large macroaggregates was lowest in the topsoil and subsoil under conventional tillage control treatment (T_1_), respectively.

### Organic carbon in soil aggregates

The topsoil had a considerably greater aggregate-associated C content across various aggregate sizes than the subsurface, as indicated in Table 2. Small macroaggregates, microaggregates, and large macroaggregates were ranked in that order, with average values of 32.73 g kg^−1^, 21.97 g kg^−1^, and 18.19 g kg^−1^, respectively. The difference in aggregate-associated C in the subsoil was less between the various aggregate sizes than it was in the topsoil. The average contents for the various treatments ranged from 10.07 to 18.85 g kg^–1^. Zero-tillage (T_5_) in the topsoil displayed a considerably greater associated C aggregate compared to rotary cultivation (T_9_) as well as conventionally tilled plots (T_12_).

**Table 2:** Big (Large) macro-aggregate-C (LMa-C), tiny (Small) macro-aggregate-C (SMa-C), as well as micro-aggregate-C (Mi-C) at both soil surface and sub-surface under various treatments during 21 years (2000–2021) of rice–wheat crop rotation study.

Treatments	LMa-C (gkg^−1^)		SMa-C (gkg^−1^)		Mi-C(gkg^−1^)	
	0-15cm	15-30cm	0-15cm	15-30cm	0-15cm	15-30cm
T_1_	12.65 ± 0.05^d^	7.38 ± 0.23	12.96 ± 0.28	11.98 ± 0.18	11.22 ± 0.03	5.78 ± 0.07
T_2_	14.11 ± 0.07^cd^	9.92 ± 0.11	21.18 ± 0.73^b^	14.12 ± 0.80^c^	23.17 ± 0.35^b^	9.36 ± 0.15
T_3_	20.35 ± 0.28^c^	10.43 ± 0.23^b^	25.51 ± 1.10^b^	19.18 ± 0.11^b^	23.62 ± 2.32^b^	11.10 ± 0.18^a^
T_4_	21.86 ± 0.17^b^	10.10 ± 0.23^bc^	56.64 ± 0.08	26.38 ± 0.23	23.18 ± 0.12^b^	11.58 ± 0.23^b^
T_5_	23.51 ± 2.47^a^	11.11 ± 0.12^ab^	58.36 ± 0.15	33.79 ± 0.03	39.42 ± 0.13	12.46 ± 0.23^b^
T_6_	9.05 ± 0.18	8.36 ± 0.15	20.42 ± 0.12^a^	13.46 ± 0.12^b^	16.05 ± 0.18	10.17 ± 0.25
T_7_	18.88 ± 0.06^d^	10.49 ± 0.58^b^	22.12 ± 0.49^d^	17.24 ± 0.37^ab^	21.25 ± 0.80^d^	12.05 ± 0.23^a^
T_8_	20.21 ± 1.09^c^	10.86 ± 0.35^b^	35.30 ± 0.86^b^	21.47 ± 0.12^b^	23.45 ± 0.35^b^	11.84 ± 0.58^b^
T_9_	20.48 ± 0.80^c^	9.92 ± 0.46^c^	53.48 ± 0.13	27.05 ± 0.18	25.25 ± 0.46^a^	11.05 ± 0.57^b^
T_10_	18.49 ± 0.09^d^	8.79 ± 0.09	19.80 ± 1.25^a^	13.19 ± 0.35^ab^	16.74 ± 0.06	7.38 ± 0.23
T_11_	18.41 ± 0.07^d^	10.35 ± 0.23^b^	23.62 ± 0.37^c^	15.80 ± 0.12^bc^	22.11 ± 1.84^c^	11.31 ± 0.12^b^
T_12_	20.39 ± 0.58^c^	11.91 ± 0.27^a^	34.76 ± 0.24^bc^	22.75 ± 0.12^a^	22.86 ± 1.04^c^	12.37 ± 0.58^a^
T_13_	21.06 ± 0.92^bc^	11.64 ± 0.12^a^	57.58 ± 0.23	27.48 ± 0.29	25.10 ± 0.58^a^	12.18 ± 0.09^a^
T_14_	15.23 ± 0.32^cd^	9.78 ± 0.07	16.46 ± 0.12^c^	12.51 ± 0.32^a^	14.11 ± 0.07	6.97 ± 0.52

In terms of aggregate-associated C, there was no discernible difference between rotary and traditional tillage. In the same tillage conditions, the associated C aggregate levels with straw return plus organic manure were considerably higher compared to those using chemical fertilizer alone. The highest aggregate-associated C was found in the zero-tillage treatment (T_5_) when it was combined with straw return and organic fertilizers. The associated C contents of the largest, smallest, and microaggregates, respectively, were 133.41%, 109.55%, and 43.31% higher than those grown under conventional tillage (T_1_), which had the lowest aggregate-related C. The aggregate-associated C concentrations in the subsoil revealed a trend of conventional tillage > rotational tillage > zero-tillage, in contrast to the topsoil. The average levels of aggregate-associated C under the T_2_, T6, and T_10_ treatments were 17.03 g kg^–1^, 13.81 g kg^–1^, and 11.45 g kg^–1^, respectively, without straw return and organic fertilizer. T_1_ was a lot higher than T_10_ and T_14_. The same tillage increased organic fertilizer and straw return aggregate-associated C concentrations in the subsurface. Microaggregates and macroaggregates of various sizes in T_10_, T_11_, T_12_, T_13_, and T_14_, respectively, had average aggregate-associated C levels of 26.37 g kg^–1^, 15.88 g kg^–1^, and 14.85 g kg^–1^ under conventional tillage. These values were much greater under organic fertilizer and straw return than they were under chemical fertilizer alone. Under the other two tillage types, comparable results were also attained.

### Water soluble carbon (WSC)

Tillage crop cultivation strategies had a significant impact on the proportion of soil mass to water stability carbon (WSC) size categories across both depths (0–15 cm and 15–30 cm). In comparison with sub-surface soil, WSC is revealed to be just 36.02% higher in surface soil (Table 3). The T_5_ treatment had the highest WSC in both depths when compared to the other treatments investigated. RT and ZT combined with organics enhanced WSC in surface soil by 9.88% and in subsurface soil by 12.4% when compared to regular tillage. T_5_ had a proportion of WSC that was noticeably higher (29.45%) than that of the other treatments when all treatments were considered. RT and ZT combined with organics enhanced WSC in surface soil by 9.88% and in subsurface soil by 12.4% when compared to regular tillage. T_5_ had a proportion of WSC that was noticeably higher (29.45%) than that of the other treatments when all treatments were considered. In soil that is on the surface and below the surface, the straw and organic fertilizer plots produced 34.78%, 29.06%, and 34.67% higher WSC than the no straw and organic fertilizer treatments ZT, RT, and CT, regardless of tillage techniques.

**Table 3:** Proportions of various carbon fractions from organic matter, cPOM and fPOM at various depths in soil as affected by tillage, straw as well as fertilizer management to the continuous RWCS.

Treatments	0–15 cm					15–30 cm				
	WSC (mg kg^−1^)	MBC (mg kg^−1^)	LFC (mg kg^−1^)	_c_POM-C (g C kg^–1^ soil)	_f_POM-C (g C kg^–1^ soil)	WSC (mg kg^−1^)	MBC (mg kg^−1^)	LFC (mg kg^−1^)	_c_POM-C (g C kg^−1^ soil)	_f_POM-C (g C kg^−1^ soil)
T_1_	17.2^e^	116.8^c^	52.7^e^	0.38^d^	0. 64^d^	13.2^e^	106.6^d^	47.9^f^	0.16^f^	0.41^f^
T_2_	25.9^c^	266.7^c^	96.4^c^	0.62^cd^	1.22^cd^	17.8^cd^	193.9^cd^	85.9^d^	0.28^de^	0.81^cd^
T_3_	28.4^d^	341.7^b^	107.8^bc^	0.79^b^	1.84^bc^	20.8^d^	219.9^bc^	96.9^cd^	0.35^cd^	1.05^bc^
T_4_	31.6^ab^	481.7^a^	160.5^a^	1.05^bc^	2.38^ab^	23.6^ab^	324.9^a^	139.7^a^	0.82a	1.93^a^
T_5_	32.5^a^	535.8^a^	183.9^a^	1.89^a^	3.78^a^	26.4^a^	361.8^a^	152.9^a^	0.92	2.34^a^
T_6_	23.9^d^	311.4^c^	91.3^c^	0.44^d^	1.03^d^	16.7^d^	187.5^cd^	66.7^e^	0.26^cd^	0.65^de^
T_7_	27.8^ab^	306.5^c^	108.1^bc^	0.62^cd^	1.82^bc^	19.6^bc^	217.8^c^	94.1^bc^	0.33^cd^	0.98^cd^
T_8_	29.2^a^	345.2^bc^	128.8^b^	0.86^bc^	2.21^ab^	21.9^bc^	260.3^b^	103.2^bc^	0.55^b^	1.33^bc^
T_9_	29.8^c^	398.6^b^	155.2^a^	1.33^ab^	2.54^a^	22.6^a^	267.3^a^	132.6^a^	0.72^a^	1.38^b^
T_10_	22.7^d^	239.9^bc^	89.2^c^	0.94^ab^	0.94^d^	17.1^cd^	166.8^cd^	65.1^d^	0.21^e^	0.59^de^
T_11_	26.4^bc^	280.7^b^	95.7^c^	0.53^cd^	1.52^cd^	18..3^ab^	196.8^bc^	87.6^c^	0.30^cd^	0.74^cd^
T_12_	29.2^cd^	343.9^ab^	123.5^b^	0.61^cd^	2.13^bc^	20.2^cd^	240.9^bc^	102.9^b^	0.44^d^	1.04^a^
T_13_	30.9^b^	424.1^a^	177.8^a^	1.30^a^	2.81^ab^	22.7^b^	294.8^ab^	141.2^a^	0.78^a^	1.64^ab^
T_14_	21.9^e^	189.2^c^	81.3^d^	0.50^c^	0.93^d^	15.1^e^	145.9^d^	49.8^e^	0.18^f^	0.48^f^

**Different letters within columns are significantly different at P = 0.05 according to Duncan Multiple Range Test (DMRT) for separation of means.

*WSC* water-soluble carbon, *MBC* microbial biomass carbon, *LFC* labile fraction carbon, *cPOM* coarse particulate organic carbon, *fPOM* fine particulate organic carbon.

### Soil microbial biomass carbon (MBC)

Between the CT and ZT without straw and organic fertilizers, the level of MBC was undetectable, and it significantly decreased below these regimens compared to ZT plus straw as well as organic fertilizers (Table 3). The cultivation strategies' impacts on the biological and biochemical qualities of the soil can be shown by changes in MBC. The fact that the MBC was higher in the ZT with straw and organic fertilizer plots than it was in the CT and RT plots under the RWCS indicates that the cropland's abandonment had a significant positive impact on microbial activity. This effect was likely brought on due to the buildup of organic C molecules in the surface soil. The fact that other labile C fractions could feed bacteria and maintain MBC when there were no plants growing is one explanation for this disparity. The condition of the soil's moisture content may also be a contributing factor. The microorganisms in the plot would be under stress during sampling in CT treatment because plant residue formation could certainly decline considerably with greater moisture in the soil (wheat maturity). CT control treatment T_1_ (111 mg kg^-1^) had the lowest average MBC when compared to other treatments.

### Light fraction of carbon (LFC)

The use of the labile fraction carbon (LFC) is thought to benefit the analysis of SOC resulting from various crop cultivation practices, such as cropping techniques and the use of both organic and synthetic fertilizer inputs. LFC in surface soil averaged 168.4, 159.5, and 143.9 mg kg^–1^ in ZT, CT, and RT treatments with straw and organics (T_5_, T_13_, and T_9_), respectively (Table 3). The growing trends in LFC content brought on by the employment of tillage techniques and residue retention in a layer of 15–30 cm was similar to those seen in a layer of 15–30 cm, but the magnitude was considerably smaller (Table 1). The CT farmers' practice (50.3 mg kg^−1^) and CT control T_1_ treatment (50.3 and 65.5 mg kg^−1^) produced the lowest average LFC when compared to other treatments, respectively.

### Particulate organic matter fractions (POM)

At the soil surface, the tillage systems' biggest variations could be seen (Table 2). Over the course of the 21-year study, the fPOM-C concentration was between 1.2 and 3 times higher under ZT with straw plus organics (T_5_) in the top 15 cm of the soil than under CT and RT with straw and organics (T_13_ and T_9_), with increases of 37.2% and 56.1%, respectively. The farmer's strategy of removing plant leftovers (straw + organics) in the ZT field explains why there was a lower concentration of cPOM-C in both soil layers with ZT, RT over CT (2.2–2.6 times less) (Table 3). These figures, with an average increase of around 101%, reflect 24 to 191% more fPOM-C when the ZT is continued with the residual. This range for cPOM was from 0.38 to 1.89, with a mean value of 0.65 on average. Under conservation tillage, particularly ZT, the tendency of decreasing OC content with depth became increasingly pronounced for both cPOM and fPOM fractions, so that the difference between average levels beneath CT and those in the 0–40 cm profile was minimal. According to Salvo et al.^40^, with continuous ZT management, the strong stratification of POM-C is typically seen as well as caused because there are no soil disturbances and agricultural residues are still present at the soil's surface. The POM-C is regarded as a critical component of soil efficiency because of its significant impact on the soil's ability to deliver nutrients and maintain structural stability in comparison to its minor total SOC contribution^41^. Two fractions of POM were found in the current investigation: fPOM and cPOM, and fPOM was, generally speaking, more responsive to land usage and soil cultivation. As opposed to fPOM, however, cPOM is more reliant on supplies of plant-derived C and, as a result, is more changeable in time, location, and depth^42^. These factors make fPOM a more trustworthy and practical indication of the effects of tillage and agricultural leftover disposal on the soil.

### Particulate organic carbon (POC)

The stratification of organic carbon in particulate form was observed in the soil. Surface soil had a higher POC than deeper soil, which decreased (Table 4). In comparison to the other crop cultivation techniques, the T_5_ (1381 mg kg^–1^), T_13_ (1156 mg kg^–1^), and T_9_ (1156 mg kg^–1^) treatments had higher POC concentrations within the soil layer of 0–15 cm. POC concentrations were higher at 0–15 and 15–30 cm in both ZT and RT with straw returns and organics than in plots with no straw and organics residue and conventionally seeded, respectively. There was less disturbance of soil macro aggregates in ZT plots, allowing more SOC to accumulate among and within the aggregation. Increased POC in ZT and RT plots relative to CT plots in the 0–15 cm and 15–30 cm soil layers is primarily due to reduced soil disturbance.

**Table 4:** Impact of 21 years of treatment implementation on the levels of different labile components of carbon in the soil.

Treatments	0–15 cm				15–30 cm			
	POC (mg kg^−1^)	PON (mg kg^−1^)	LFOC (mg kg^−1^)	LFON (mg kg^−1^)	POC (mg kg^−1^)	PON (mg kg^−1^)	LFOC (mg kg^−1^)	LFON (mg kg^−1^)
T_1_	620^d^	22.5^ef^	52.7^e^	5.2^d^	485^e^	41.5^f^	42.8^e^	5.2^e^
T_2_	788^cd^	72.9^d^	91.3^c^	8.9^d^	674^cd^	71.5^cd^	84.1^cd^	8.1^cd^
T_3_	1033^b^	97.4^bc^	107.8^bc^	11.8^c^	813^a^	79.4^c^	96.9^bc^	9.6^bc^
T_4_	1357^a^	126.7^ab^	155.2^a^	13.3^ab^	942^ab^	106.1^a^	142.9^a^	11.4^a^
T_5_	1381^a^	130.8^a^	183.9^a^	13.8^a^	1032^a^	112.1^a^	152.9^a^	12.4^a^
T_6_	779^cd^	69.9^d^	81.3^d^	8.1^d^	609^de^	69.1^de^	81.6^cd^	7.9^de^
T_7_	898^bc^	92.6^cd^	96.4^c^	10.5^bc^	785^bc^	73.3^cd^	91.6^bc^	8.9^bc^
T_8_	1102^b^	103.9^bc^	123.5^b^	11.5^b^	886^b^	91.8^ab^	103.2^b^	10.9^ab^
T_9_	1156^ab^	114.2^ab^	160.5^b^	12.6^ab^	905^ab^	96.7^ab^	139.7^a^	11.9^a^
T_10_	638^d^	67.2^d^	78.2^cd^	7.6^d^	585^c^	67.3^e^	77.9^de^	7.1d^e^
T_11_	869^c^	88.5^c^	95.7^c^	9.5^c^	728^b^	72.8^cd^	86.7^cd^	8.2^cd^
T_12_	1056^bc^	98.8^c^	108.1^bc^	12.6^bc^	789^b^	86.5^cd^	97.9^b^	9.9^bc^
T_13_	1285^a^	117.5^a^	128.8^b^	14.2^a^	974^a^	103.3^b^	141.2^a^	11.8^a^
T_14_	631^d^	44.7^ef^	89.2^c^	6.8^d^	535^e^	54.7^f^	65.1^de^	6.8^e^

Values in a column followed by the same letter are not significantly different (P < 0.05).

*POC* Particulate organic carbon, *PON* Particulate organic nitrogen, *LFOC* Light fraction organic carbon, *LFON* Light fraction organic nitrogen.

This occurrence may lead to a long-term stabilization of SOC concealed within certain micro-aggregates, which could also result in the creation of microaggregates within macro-aggregates created around fine intra-aggregate POC. The results of this study show that ZT and RT have a significant influence on the formation and stabilization of SOM only in the 0–15 cm soil layer, and that after 21 years of rice–wheat cropping in *Typic ustothrepts*, soil-altered organic matter besides residue decay contained significantly higher POC in the 0–15 cm than in the chemically reactive fertilizer treatment options. POC elevation is considered a potential sign of elevated C accumulation^43^.

### Particulate organic nitrogen (PON)

Table 3 shows the amount of particulate organic nitrogen (PON) across the CT (T_6_) of the field following a 21-year crop cycle. The PON change in the top and below depths (0–15 and 15–30 cm) varied significantly. Over a 21-year study period, ZT with straw and organic (T_5_) plots had the highest PON change (68%) compared to CT with farmers' practices (T_12_), which was followed by RT with straw and organic (T_13_) plots (53.4%) and RT with straw and organic (T_9_) plots (46%) In comparison to other treatments, CT control treatment T_1_ (32.0 mg kg^-1^) had the lowest average PON. Similar growth tendencies were seen at lower depths (15–30 cm), but the magnitude was much smaller (Table 3).

### Organic carbon with a labile fraction (LFOC) and nitrogen (LFON)

A valuable method for characterizing SOC resulting from various soil management activities, such as tillage techniques, cropping systems, and the application of fertilizer sources, is the labile fraction of organic carbon (LFOC). The average values of LFOC in surface soil in ZT and RT with straw and organic (T_5_, T_9_) and CT with straw and organic (T_13_), respectively, were 168.4, 150.1, and 135.0 mg kg^–1^ (Table 4). The CT control treatment T_1_ (47.75 mg kg^–1^) had the lowest LFOC in comparison to other treatments Similar to those appearing in the layer of 0–15 cm, but with a smaller magnitude, the growing trends in LFOC content caused by the application of tillage techniques with straw and organics were seen in the 15–30 cm layer (Table 2). Results from a 21-year trial on LFON content revealed that T_13_, T_5_, and T_9_ treatments raised the LFON composition of the tillage system's 0–15 cm soil layer from 13.8 mg kg^-1^ in CT to 13.8, and 12.6 mg kg^–1^ with straw and organics under ZT and RT, respectively (Table 3). Similar to those seen in the 0–15 cm layer, although with a smaller magnitude, were the growing trends in LFON concentration brought on by the application of tillage and residue management techniques in the 15–30 cm layer (Table 3). In comparison to CT farmers' practices (T_14_) and the unfertilized CT control plot (T_1_), all of the treatments receiving inorganic fertilizer (T_2_, T_6_, T_11_) maintained significantly higher levels of LFON content in the surface soil (Table 2).

### Characteristics of the soil biology

#### Potentially mineralizable nitrogen (PMN)

Over the course of the 21-year experiment, the layer of 0–15 cm in the T_5_ (14.6 mg kg^–1^), T_13_ (12.6 mg kg^–1^), and T_9_ (11.6 mg kg^–1^) treatments showed the highest levels of potentially mineralizable nitrogen (PMN) content. In comparison to the no-fertilizer (T_1_) and fertilizer-alone (T_2_, T_6_, T_10_) treatments, the usage of organic fertilizer plus the return of straw enhanced the PMN under the ZT, RT, and CT tillage methods. The CT control treatment T_1_ (3.3 mg kg^–1^) produced the lowest PMN in comparison to other treatments (Table 5). The tillage crop cultivation techniques' growth tendencies in the layer of 15–30 cm was similar to those in the layer of 0–15 cm, but their magnitude was noticeably smaller (Table 4).

**Table 5:** Impact of 21 years of treatment application on the levels of different biological carbon components in the soil.

Treatments	0–15 cm				15–30 cm			
	PMN (mg kg^−1^)	MBC (mg kg^−1^)	MBN (mg kg^−1^)	DOC (mg kg^−1^)	PMN (mg kg^−1^)	MBC (mg kg^−1^)	MBN (mg kg^−1^)	DOC (mg kg^−1^)
T_1_	3.3^e^	106.7^f^	5.1^e^	89.9^f^	2.9^e^	85.9^e^	4.5^f^	82.8^ef^
T_2_	8.3^cd^	319.2^cd^	11.9^de^	128.3^de^	6.9^de^	196.8^cd^	10.3^de^	106.9^de^
T_3_	10.3^bc^	418.6^bc^	19.9^cd^	144.1^bc^	7.8^bc^	296.9^bc^	13.1^cd^	121.2^cd^
T_4_	12.4^a^	517.7^b^	27.1^a^	193.6^a^	11.9^a^	409.3^a^	19.9^a^	151.1^a^
T_5_	14.6^a^	526.2^a^	35.8^a^	212.8^a^	13.9	424.9^a^	25.6^a^	161.9^a^
T_6_	7.6^cd^	279.5^de^	9.8^de^	122.5^de^	6.6^de^	187.5^cd^	9.5^ef^	104.6^de^
T_7_	9.9^cd^	389.9^cd^	16.7^cd^	136.4^cd^	7.4^cd^	256.8^cd^	12.8^cd^	119.6^cd^
T_8_	11.6^ab^	481.7^a^	22.7^ab^	166.4^ab^	9.9^ab^	354.8^ab^	14.9^ab^	138.6^ab^
T_9_	12.5^a^	493.9^ab^	25.4^ab^	187.9^ab^	10.4^ab^	367.3^ab^	18.5^ab^	146.3^ab^
T_10_	6.7^cd^	261.4^de^	9.1^de^	113.5^de^	5.9^de^	173.9^cd^	8.7^ef^	101.7^de^
T_11_	9.5^cd^	345.2^cd^	14.9^cd^	126.9^cd^	6.6^cd^	219.8^cd^	11.8^cd^	112.9^cd^
T_12_	10.8^bc^	470.7^bc^	21.1^bc^	155.7^bc^	8.2^bc^	319.9^bc^	13.9^bc^	126.4^bc^
T_13_	12.6^a^	515.8^a^	26.3^a^	197.6^a^	11.2^a^	401.8^a^	19.6^a^	148.6^ab^
T_14_	5.6^e^	196.8^e^	7.7^e^	103.7^e^	3.8^e^	106.6^de^	6.1^f^	92.3^ef^

Values in a column followed by the same letter are not significantly different (P < 0.05).

*PMN* potentially mineralizable Nitrogen, *MBC* Soil microbial biomass carbon, *MBN* Soil microbial biomass nitrogen, and *DOC* Dissolved organic carbon.

#### Carbon via microbial biomass (MBC)

When compared to the ZT and RT plus straw returns and organic fertilizer, without straw returns, there was no way to tell the difference between the CT and ZT in the quantity of MBC, and it was substantially less under certain regimes. (Table 5). The cultivation strategies' impacts on biological and biochemical soil peculiarities can be shown by changes in MBC. The fact that MBC was found to be higher in both ZT and RT plots makes it clear that cropland abandonment had a major favourable impact on the activities of microbial species since residue retention was higher in the abandoned plot compared to the CT plot containing wheat crops, which were probably brought on by the buildup of organic C compounds at the soil surface. When compared to other treatments, the CT control treatment T1 in the 0–15 and 15–30 cm layers had the lowest MBCs (106.7 and 85.9 mg kg^–1^) (Table 3). The fact that other labile C fractions could feed bacteria and maintain MBC in the absence of growing plants is one explanation for this disparity. The condition of the soil's moisture content may also be a contributing factor. The microorganisms in the plot would be under stress during sampling in CT plots as organic matter development might undoubtedly significantly reduce soil water (wheat maturity).

#### Microbial nitrogen biomass (MBN)

Following 21 years, analysis of MBN material showed that T_5_ (35.8 mg kg^–1^), T_13_ (26.3 mg kg^–1^), and T_9_ (25.4 mg kg^–1^) treatments were superior to the other treatments in the 0–15 cm soil layer. Straw plus organic fertilizers enhanced the MBN content in ZT, CT, and RT compared to no organic fertilizers and chemical fertilizers alone treatments (Table 5). Similar to those seen in the 0–15 cm layer, although with a smaller magnitude, were the growing trends in MBN content brought on by the employment of tillage procedures with straw and organics in the 15–30 cm layer (Table 4). In plots receiving T_5_, T_9_, T_13_, and over CT farmers' practice plots (T_14_) and CT control plots (T_1_), respectively, MBN within the soil surface (0–15 cm) continued to significantly rise. (Table 6). Additionally, in a layer between 15 and 30 cm, the similarities in trends brought on by crop cultivation techniques involving tillage were contrasted with those of the CT "control" plot.

**Table 6:** Variations in the soil organic carbon (SOC) content (g kg-1) during 21 years of study as influenced by tillage, straw, and fertilizer management practices (standard deviation from the mean) (At the conclusion of the experiment in 2021).

Soil Depth (Cm)	Initial 2000	T_1_	T_2_	T_3_	T_4_	T_5_	T_6_	T_7_
0–15	4.7 ± 0.26	2.6 ± 0.12^b^	3.1 ± 0.19^b^	3.9 ± 0.18^b^	5.4 ± 0.26^a^	5.8 ± 0.28^a^	3.0 ± 0.17^b^	3.7 ± 0.19^b‡^
15–30	4.5 ± 0.25	2.0 ± 0.09^b^	2.7 ± 0.12^b^	3.2 ± 0.17^a^	5.2 ± 0.22^a^	5.5 ± 0.23^a^	2.5 ± 0.11^b^	3.1 ± 0.18^c^
30–60	3.1 ± 0.19	1.8 ± 0.10^ab^	2.3 ± 0.15^ab^	2.4 ± 0.13^a^	4.5 ± 0.19^b^	5.1 ± 0.21^b^	2.1 ± 0.13^ab^	2.1 ± 0.13^d^
60–90	2.3 ± 0.13	0.9 ± 0.05^b^	1.2 ± 0.12^b^	1.9 ± 0.11^a^	2.7 ± 0.15^a^	3.4 ± 0.19^c^	1.1 ± 0.10^b^	1.1 ± 0.07^b^
90–120	1.4 ± 0.09	0.69 ± 0.04^c^	0.93 ± 0.06^c^	1.1 ± 0.07^b^	1.9 ± 0.12^b^	2.3 ± 0.13^d^	0.87 ± 0.06^c^	0.9 ± 0.05^c^
Mean	3.2 ± 0.18	1.6 ± 0.08^b^	2.1 ± 0.13^ab^	2.5 ± 0.13^b^	3.9 ± 0.19 ^b^	4.4 ± 0.21^a^	1.9 ± 0.11^b^	2.2 ± 0.12^c^

Soil Depth (Cm)	Initial 2000	T_8_	T_9_	T_10_	T_11_	T_12_	T_13_	T_14_
0–15	4.7 ± 0.26	4.8 ± 0.23^b^	4.9 ± 0.23^a^	2.9 ± 0.16^b^	3.4 ± 0.22^b^	4.4 ± 0.24^ab^	5.1 ± 0.21^b^	2.7 ± 0.14^b^
15–30	4.5 ± 0.25	3.3 ± 0.18^c^	4.1 ± 0.21^b^	2.4 ± 0.11^b^	2.9 ± 0.14^b^	3.1 ± 0.12^bc^	4.6 ± 0.19^b^	2.2 ± 0.10^b^
30–60	3.1 ± 0.19	2.8 ± 0.15^c^	3.1 ± 0.18^c^	2.1 ± 0.12^ab^	2.4 ± 0.18^ab^	2.6 ± 0.11^c^	3.3 ± 0.18^c^	2.0 ± 0.11^ab^
60–90	2.3 ± 0.13	1.9 ± 0.11^a^	2.3 ± 0.14^a^	1.0 ± 0.08^b^	1.3 ± 0.16^b^	1.5 ± 0.10^b^	2.8 ± 015^d^	1.0 ± 0.06^b^
90–120	1.4 ± 0.09	1.2 ± 0.07^b^	1.5 ± 0.09^b^	0.82 ± 0.05^c^	0.98 ± 0.06^c^	1.1 ± 0.0.09^cd^	1.6 ± 0.10^b^	0.78 ± 0.05^c^
Mean	3.2 ± 0.18	2.8 ± 015^c^	3.2 ± 0.17^c^	1.8 ± 0.10^b^	2.2 ± 0.15^b^	2.5 ± 0.17^c^	3.5 ± 0.18^b^	1.7 ± 0.09^b^

**Different letters within columns are significantly different at P = 0.05 according to Duncan Multiple Range Test (DMRT) for separation of means.

#### Organic carbon dissolved (DOC)

On Table 5, the amount of organic carbon dissolved (DOC) over a field's CT (T_1_) following a 21-year crop cycle is shown. Different DOC changes were seen in the upper and lower soil layers (0–15 and 15–30 cm, respectively). In comparison to the other treatments, the T_5_ (212.8 mg kg^–1^), T13 (197.6 mg kg^–1^), and T_9_ (187.9 mg kg^–1^) treatments showed the highest DOC change. The use of ZT, RT, and CT with organics boosted DOC by 64.2% more than that of ZT, RT, and CT without organics (T_5_, T_9_, and T_13_) plots for the rice–wheat crop cycle, while the conventional tillage with farmers' practices (T_14_) and CT control (T_1_) plots had the lowest DOC, respectively.

### Soil organic carbon (SOC) patterns

Between treatments and depths, significant variations in SOC levels have been noticed. (p£0.05). (Table 6). At all soil levels (0–15, 15–30, 60–90, and 90–120 cm), T_5_ had the greatest SOC content with 5.8 g kg^–1^ in the upper layer (0–15 cm), followed by T_13_ (5.1 g kg^–1^) and T_9_ (4.8 g kg^–1^) treatments (Table 6). In comparison to those not receiving any organic fertilizers, SOC contents in subsurface and surface regions were greater in all plots fertilized using organic fertilizers. T_4_ (5.4 g kg^–1^), T_8_ (4.7 g kg^–1^), and T_5_ treatments increased the SOC concentration (4.4 g kg^–1^). However, even in the subsoil, the SOC content rose in response to the application of organic materials (Table 5). The mean profile SOC content increased to 5.8 g kg^–1^ in T_5_ from 1.7 g kg^–1^ in T_14_. However, in treatments T_2_, T_6_, and T_10_, there was no rise in SOC concentration. It is well known that applying the same amount of nutrients as chemical fertilizers does not increase the SOC concentration as much as applying organic manures and compost does^44^. After a change in management practice, tillage systems were found to cause disparities in SOC that started in the third year and grew more pronounced in the following years. The control treatment (CT) had the lowest SOC concentrations in various soil depths throughout the course of 21 years, averaging 1.6 g kg^–1^ in comparison to other treatments.

### SOC pools, carbon budgeting, and C sequestration

Table 9 shows the field's capacity for sequestering carbon through tillage crop cultivation practices after sixteen crop cycles. Following CT with straw returns and organic fertilizer (T_13_) and RT with straw returns and organic fertilizer (T_9_), respectively, ZT with straw and organic fertilizer (T_5_) has recorded the highest SOC pool equivalent depth and mass basis (36.23 and 34.30 Mg ha^−1^), potential for sequestering carbon dioxide (9.76 Mg C ha^−1^), rate of C sequestration (1.19 Mg C ha^−1^ yr^−1^) and efficiency of C sequestration (35.81%) (Table 7). Zero tillage has a higher potential for sequestering carbon than rotary tillage and conventional tillage treatment combinations by 21.5% and 23.2%, respectively. The addition of organic fertilizer and straw return improved the SOC pools, C sequestration potential, C sequestration rate, and C sequestration efficiency under the ZT, RT, and CT tillage methods, respectively. Compared to the other treatments, CT with no chemical fertilizers (T_1_) has the lowest potential for C sequestration.

**Table 7:** Impact of tillage, crop cultivation practices, and fertilizer management strategies on carbon budget and carbon sequestration of soil profile over 21 years of RWCS.

Treatments	Equivalent depth basis SOC pool (0–30 cm) (Mg ha^–1^)	Equivalent mass basis SOC pool (0–30 cm) (Mgha^–1^)	Potential C sequestration (Mg C ha^−1^)	C sequestration rate (Mg C ha^−1^ yr^−1^)	Efficiency of C sequestration (%)
T_1_	11.35^e^	10.67^e^	0.67^e^	0.12^e^	1.28^d^
T_2_	24.80^d^	26.18^d^	2.53^d^	0.39^e^	9.78^a^
T_3_	29.54^bc^	28.85^cd^	3.33^b^	0.49^a^	13.34^ab^
T_4_	33.59^ab^	31.82^ab^	4.83^bc^	0.63^ab^	19.46^a^
T_5_	36.23^a^	34.30^a^	9.76 ^a^	1.19^a^	35.81^a^
T_6_	24.23^d^	24.26^e^	2.09^b^	0.37^ab^	9.20^d^
T_7_	25.26^cd^	26.96^cd^	3.03^b^	0.46^b^	11.51^bc^
T_8_	32.81^ab^	30.29^ab^	4.77^c^	0.57^bc^	18.10^c^
T_9_	35.24^ab^	31.69^ab^	6.93 ^ab^	0.82^ab^	28.43^ab^
T_10_	24.15^d^	24.16^cd^	1.87^cd^	0.36^e^	8.82^a^
T_11_	24.96^d^	26.31^d^	3.01^bc^	0.44^de^	10.23^ab^
T_12_	30.76^bc^	30.17^bc^	3.89^b^	0.54^cd^	16.37^a^
T_13_	36.09^a^	34.02^ab^	6.87^a^	0.78^a^	27.16^b^
T_14_	23.82^d^	21.69^d^	0.87^e^	0.22^d^	6.53^cd^

**Different letters within columns are significantly different at P = 0.05 according to Duncan Multiple Range Test (DMRT) for separation of means.

### SOC changes over time: a comparative analysis

In all of the investigated soil phases, tillage crop cultivation techniques had a significant impact on SOC when measured during the past 21 years (Table 8). Under the tested T_1_, T_14_, T_10_, T_6_, T_2_, T_7_, and T_11_ treatments, SOC concentrations declined between 0 and 400 kg of soil m^−2^. Stocks of SOC in the top 400 kg of soil m^−2^ decreased from 3.77 to 3.48 kg of C m^−2^ representing a change of − 0.09 ± 0.2 kg of C m^−2^ in T1, 4.07 to 3.98 kg of C m^−2^ represented a change of − 0.19 ± 0.2 kg of C m^−2^ in T14, 4.47 to 4.18 kg of C m^−2^ represented a change of − 0.29 ± 0.2 kg of C m^−2^ in T10, 4.97 to 4.38 kg of C m^−2^ represented a change of − 0.39 ± 0.2kg of C m^−2^ in T6, 5.77 to 4.98 kg of C m^-2^ represented a change of -0.49 ± 0.2 kg of C m^−2^ in T2, 6.92 to 6.22 kg of C m^-2^ represented a change of − 0.70 ± 0.09 kg of C m^−2^ in T7, 5.92 to 5.22 kg of C m^−2^ represented a change of − 0.70 ± 0.09 kg of C m^−2^ in T11, over 2000 to 2021 (Table 7). Present findings unequivocally demonstrate the fact that zero-tillage and rotary tillage treatments, when combined with straw returns and organic fertilizers, are capable of capturing carbon dioxide from the atmosphere or even achieving equilibrium among inputs and outcomes under the study's specific conditions (climate, soil type, tillage system with straw and organic fertilizer). After 21 years of agriculture without straw returns and organic fertilizers under zero tillage, rotary tillage, and conventional tillage approaches, levels of SOC were obviously lower, and further study will be required to identify whether and once the system has achieved equilibrium or a constant state. After 21 years, there was a similar shift in the soil C content in the intervals of 400–800 and 800–1200 kg of soil m^–2^. Averaged variations in tillage crop cultivation techniques during the course of the research, CT without straw returns and chemical fertilizers, were –0.11 ± 0.2 and –0.02 ± 0.1 kg C m^−2^ in the intervals of 400–800 and 800^−1^ 200 kg of soil per m^2^. Chemical fertilizers and straw returns were not used. For each of the indicated soil mass periods, this translates to an estimated yearly frequency of –36.6 and –22.9 g C m^−2^ yr^−1^. (Table 7). This SOC inventory was modest and well below the precision of our assessment at the 400–800 as well as 800–1200 kg of soil m^–2^ intervals, given the uncertainty associated with these estimations.

**Table 8:** Stocks of soil organic carbon (SOC) and the annualized rate of change in various soil mass intervals throughout 2000 until 2021 (adjusted for tillage, crop residue management, and fertilizer application rate).

Treatments	Soil Organic Carbon, kgm^−2^ (± Standard error)											
	0–400 kg of soil m^−2^ (approx. 0–30 cm)			Annual SOC change rate g of Cm^–2^ yr^−1^	400–800 kg of soil m^−2^ (approx. 30–60 cm)			Annual SOC change rate g of Cm^–2^ yr^−1^	800–1200 kg of soil m^−2^ (approx. 60–90 cm)			Annual SOC change rate g of Cm^–2^ yr^−1^
	2000	2021	Difference		2000	2021	Difference		2000	2021	Difference	
**T**_**1**_	3.77	3.48	− 0.09 ± 0.2	− 48.2	2.92	2.31	− 0.11 ± 0.2	− 36.6	2.09	1.98	− 0.02 ± 0.1	− 22.9
**T**_**2**_	5.77	4.98	− 0.49 ± 0.2	− 26.2	4.02	3.31	− 0.31 ± 0.2	− 11.6	2.74	2.12	− 0.02 ± 0.1	− 8.9
**T**_**3**_	7.46	7.15*	0.31 ± 0.03	28.2	5.39	5.65	0.26 ± 0.09	6.9	3.14	3.12	− 0.02 ± 0.01	− 1.8
**T**_**4**_	8.12	9.11*	0.99 ± 0.2	82.1	5.47	5.57	0.10 ± 0.09	8.8	3.38	3.47	0.01 ± 0.11	5.4
**T**_**5**_	8.98*	9.77	0.79 ± 0.2	66.2	7.03	7.11	0.08 ± 0.2	9.5	3.72	3.81	0.09 ± 0.11	8.1
**T**_**6**_	4.97	4.38	− 0.39 ± 0.2	− 31.2	3.82	3.21	− 0.28 ± 0.2	− 17.6	2.34	2.03	− 0.02 ± 0.1	− 11.9
**T**_**7**_	6.92	6.22	− 0.70 ± 0.09	− 13.4	5.05	4.98	− 0.07 ± 0.09	− 5.5	3.42	3.37	− 0.05 ± 0.02	− 3.9
**T**_**8**_	8.81	8.75	0.06 ± 0.05	25.7	5.82	5.31*	0.51 ± 0.2	4.5	2.93	2.67	0.26 ± 0.02	1.9
**T**_**9**_	9.18*	9.87	0.69 ± 0.2	57.4	7.62	7.64	0.02 ± 0.2	7.0	5.04	5.08	0.04 ± 0.01	3.7
**T**_**10**_	4.47	4.18	− 0.29 ± 0.2	− 36.2	3.12	3.06	− 0.21 ± 0.2	− 19.6	2.44	2.09	− 0.02 ± 0.1	− 17.9
**T**_**11**_	5.92	5.22	− 0.70 ± 0.09	− 16.4	4.05	3.98	− 0.07 ± 0.09	− 8.5	2.82	2.17	− 0.05 ± 0.02	− 7.9
**T**_**12**_	8.16	8.65	0.21 ± 0.03	18.2	5.27	5.47	− 0.10 ± 0.09	3.8	3.11	3.62	0.09 ± 0.11	2.1
**T**_**13**_	9.15	9.29	0.14 ± 0.9	19.6	5.72	5.88	0.16 ± 0.09	7.3	4.57	4.58	0.01 ± 0.01	3.6
**T**_**14**_	4.07	3.98	− 0.19 ± 0.2	− 46.2	3.02	2.91	− 0.18 ± 0.2	− 31.6	2.24	2.03	− 0.02 ± 0.1	− 19.9

*Significant difference between years at α = 0.05.

The tillage crop cultivation procedures (zero tillage, rotary tillage, as well as conventionally tilled treatments) had a substantial impact on stocks of SOC in the 2000–2021 samples when SOC variations through time were taken into account overall for 1200 kg of soil m^–2^ (Table 7). SOC increased with the usage of organic fertilizers, while it decreased when they were not used. These variations in SOC were detected depending on the treatment combinations. Under ZT, SOC changed from 12.55 to 10.79 (T_2_), 14.96 to 14.13, (T_3_), 20.79 to 21.55 (T_4_), 22.33 to 24.31 (T_5_) kg of C m^–2^ between 2000 and 2021, rotary tillage 11.07 to 10.15 (T_6_), 13.14 to 12.70 (T_7_), 18.07 to 16.55 (T_8_), 21.70 to 22.44 (T_9_) and under CT from 10.14 to 9.40 (T_10_), 11.65 to 10.79 (T_11_), 16.85 to 16.35 (T_12_), 20.89 to 21.86 (T_13_) and 09.10 to 8.20 (T_14_) and greater decreased found in 08.75 to 7.70 (T_1_) kg of C m^–2^ over 2000 to 2021 respectively. The rate of SOC loss with CT occurred at a pace that has been 1.1 times faster than ZT, and it followed a similar trend to the rotary tillage treatments, according to archived data (Table 9), even despite the existence of a scientifically substantial distinction. The research findings highlight the importance of archiving samples in order to assess the long-term effects of agricultural cultivation techniques on SOC^45^. SOC stores in 1200 kg (approx. 0–90 cm) of soil m^–2^ averaged under intensive crop farming practices declined by -0.93 ± 0.4 kg m^–2^ from 10.14 to 9.40 (T_10_) kg m^–2^, -0.66 ± 0.4 kg m^–2^ from 11.65 to 10.79 (T_11_) kg m^–2^ and -0.96 ± 0.4 kg m^-2^ from 09.10 to 8.20 kg m^–2^ (T_14_) in 2000 to 2016, while both ZT and RT plus residual retaining reserves of SOC about 1200 kg of soil m^–2^ grew by 1.98 ± 0.03* kg m^–2^ from 22.33 to 24.31 (T_5_) kg of C m^–2^ in ZT and 1.52 ± 0.4 kg m^–2^ from 18.07 to 16.55 (T_8_) kg of C m^–2^ in rotary tillage treatments combinations with organic fertilizers (Table 8). Despite the fact that trends indicate that C has been lost from soil, but rather than being captured from the atmosphere, it has been captured from the soil, the amount of SOC in the 1200 kg of soil m^–2^ period between 2000 and 2021 was not particularly notable.

**Table 9:** Stocks of soil organic carbon (SOC) (0–90 cm), system effectiveness, and energy usage pattern in relation to management of tillage crop residue and nutrient strategies to continuous RWCS.

Treatments	Soil Organic Carbon, kgm^-2^ (± Standard error)				Total input Energy (GJha^–1^)	Specific energy (MJha^–1^)	Energy use efficiency	Net energy (GJ ha^–1^)	Total field efficiency (%)
	0-1200kg of soil m^–2^ (approx. 0–90 cm)			Annual SOC change rate g of Cm^–2^ yr^–1^					
	2000	2021	Difference						
**T**_**1**_	8.75	7.70	− 0.98 ± 0.4	− 71.7	32.8	7.98	4.35	64.6	48.15
**T**_**2**_	12.55	10.79	− 0.26 ± 0.4	− 33.3	30.3	6.35	5.45	63.2	49.95
**T**_**3**_	14.96	14.13	0.83 ± 0.2*	31.3	28.9	5.49	6.15	61.7	51.98
**T**_**4**_	20.79	21.55	0.76 ± 0.4	63.3	25.7	4.60	6.90	50.1	65.06
**T**_**5**_	22.33	24.31	1.98 ± 0.03*	99.2	21.2	3.91	7.42	42.9	81.44
**T**_**6**_	11.07	10.15	− 1.22 ± 0.4	− 26.7	30.9	6.75	5.15	63.5	49.35
**T**_**7**_	13.14	12.70	− 0.33 ± 0.4	− 31.7	29.2	5.99	6.05	62.1	50.65
**T**_**8**_	18.07	16.55	1.52 ± 0.4	26.7	26.4	4.97	6.70	50.8	60.13
**T**_**9**_	21.70	22.44	0.74 ± 0.4	61.7	23.1	3.95	7.12	46.4	76.16
**T**_**10**_	10.14	9.40	− 0.93 ± 0.4	− 41.7	31.7	7.15	4.85	63.8	49.15
**T**_**11**_	11.65	10.79	− 0.66 ± 0.4	− 38.3	29.8	6.12	5.95	62.9	50.15
**T**_**12**_	16.85	16.35	0.50 ± 0.22	15.5	23.8	4.05	7.04	46.8	73.25
**T**_**13**_	20.89	21.86	0.97 ± 0.2*	79.2	24.6	4.45	7.08	48.3	66.71
**T**_**14**_	9.10	8.20	− 0.96 ± 0.4	− 51.7	32.2	7.65	4.55	64.1	48.35

*Significant difference between years at α = 0.05.

### Efficiency of energy usage and dynamics

In view of the current energy crisis, research on energy dynamics and energy usage efficiency in agroecosystems assumes enormous importance in order to pinpoint viable production systems that rely less on non-renewable fossil fuels. The calculation of energy use in various tillage crop residue techniques in the current study indicated that T_1_ (conventionally tilled control plot), T_14_ (conventional tillage farmers plot) and T_10_ (conventional tillage coupled with chemical fertilizer plots) utilized highest energy (32.8, 32.2 and 31.7 GJ ha^–1^) followed by T_6_ (rotary tillage coupled with chemical fertilizer plots) (30.9 GJ ha^–1^), and T_2_ (zero tillage coupled with chemical fertilizer plots) (30.3 GJ ha^–1^) over all the other treatment combinations with straw returns, organic fertilizer and chemical fertilizers of conventional, rotary and zero tillage found to be lowest energy use (T_3_ to T_5_; T_7_ to T_9_, T_11_ to T_13_, respectively (Table 9). The highest energy input was used in T_1_, T_10_, T_14_, and T_6_ (conventional tillage practices) because rice requires more energy for transplanting, thrashing, and nursery-raising operations than wheat does. Rice also requires more energy input during tillage operations than wheat due to the puddling, nursery raising, and labor-intensive transplanting and thrashing processes. Due to the need for regular herbicide spraying on rice crops due to their propensity for weed infestation and the frequent irrigation requirements for rice and wheat, T_6_ (rotary tillage) and T_2_ (zero tillage) both require more energy than other tillage methods^46,47^.

## Conclusions

This long-term experiment concludes that by altering the aggregate and distribution of C therein, tillage techniques can alter the dynamics of microbial biomass in soils and organic soil carbon. Conservation tillage (zero-till combined with the return of straw and organic fertilizers) improved water stability by creating large macro-aggregates and micro-aggregates in contrast to traditional tillage under both the surface as well as the subsurface soil. Using tillage strategies for conservation methods, the stability of large macro-aggregates (> 2 mm), small macro-aggregates (2.0–2.25 mm), and micro-aggregates in the topsoil was improved by 35.18%, 33.52%, and 25.10%, respectively, over conventional tillage (0–20 cm). The 20–40 cm subsoil showed the similar pattern. When conservation tillage with organic and chemical fertilizers was applied, macro-aggregates of all sizes and micro-aggregates rose by 24.52%, 28.48%, and 18.12%, respectively, in contrasting to conventional tilling with no straw returns. Aggregation improved under conservation tillage utilizing straw as opposed to conventional tillage without straw return due to its mulching effects and the addition of organic matter in long-term studies like the present one. Because of the straw return, the aggregate-associated carbon level increased significantly. The overall aggregate-associated C in the topsoil was higher across all aggregate proportions when zero tillage was combined with a straw return than in subsurface soils when conventional tillage with a straw return was used.

## Data Availability

The datasets used and/or analysed during the current study are available from the corresponding author on reasonable request.
