# Publisher Correction: Long‑term application of agronomic management strategies effects on soil organic carbon, energy budgeting, and carbon footprint under rice–wheat cropping system

**DOI:** 10.1038/s41598-024-56776-x

**Published:** 2024-03-15

**Authors:** R. K. Naresh, P. K. Singh, Rajan Bhatt, Mandapelli Sharath Chandra, Yogesh Kumar, N. C. Mahajan, S. K. Gupta, Nadhir Al-Ansari, Mohamed A. Mattar

**Affiliations:** 1 Department of Agronomy, Sardar Vallabhbhai Patel University of Agriculture & Technology, Meerut, UP India; 2 Director Extension Education, Sardar Vallabhbhai Patel University of Agriculture & Technology, Meerut, UP India; 3 Krishi Vigyan Kendra, Amritsar, Punjab Agricultural University, Ludhiana, Punjab India; 4 AICRP On Integrated Farming System, Professor Jayashankar Telangana State Agricultural University, Rajendranagar, Telangana India; 5 Department of Soil Science & Agricultural Chemistry, Sardar Vallabhbhai Patel University of Agriculture & Technology, Meerut, UP India; 6 Institute of Agricultural Science, Department of Agronomy, Banaras Hindu University, Varanasi, U. P India; 7 Department of Agronomy, Bihar Agricultural University Sabour, Bhagalpur, Bihar India; 8 Department of Civil, Environmental and Natural Resources Engineering, Lulea University of Technology, 97187 Lulea, Sweden; 9 Prince Sultan Bin Abdulaziz International Prize for Water Chair, Prince Sultan Institute for Environmental, Water and Desert Research, King Saud University, 11451 Riyadh, Saudi Arabia; 10 Department of Agricultural Engineering, College of Food and Agriculture Sciences, King Saud University, 11451 Riyadh, Saudi Arabia

Correction to: *Scientific Reports* 10.1038/s41598-023-48785-z, Published online 03 January 2024

In the original version of this Article, Mohamed Mattar was omitted as a corresponding author. Correspondence and requests for materials should also be addressed to mmattar@ksu.edu.sa.

